# Desmin enters the nucleus of cardiac stem cells and modulates Nkx2.5 expression by participating in transcription factor complexes that interact with the *nkx2.5* gene

**DOI:** 10.1242/bio.014993

**Published:** 2016-01-19

**Authors:** Christiane Fuchs, Sonja Gawlas, Philipp Heher, Sofia Nikouli, Hannah Paar, Mario Ivankovic, Martina Schultheis, Julia Klammer, Teresa Gottschamel, Yassemi Capetanaki, Georg Weitzer

**Affiliations:** 1 Department of Medical Biochemistry, Max F. Perutz Laboratories, Medical University of Vienna, Vienna Biocenter, Vienna A1030, Austria; 2 Center of Basic Research, Biomedical Research Foundation, Academy of Athens, Athens 115 27, Greece

**Keywords:** Cardiac progenitor cells, Desmin, Nkx2.5, Nuclear localization, Transcriptional regulation, Intermediate filament protein

## Abstract

The transcription factor Nkx2.5 and the intermediate filament protein desmin are simultaneously expressed in cardiac progenitor cells during commitment of primitive mesoderm to the cardiomyogenic lineage. Up-regulation of Nkx2.5 expression by desmin suggests that desmin may contribute to cardiogenic commitment and myocardial differentiation by directly influencing the transcription of the *nkx2.5* gene in cardiac progenitor cells. Here, we demonstrate that desmin activates transcription of *nkx2.5* reporter genes, rescues *nkx2.5* haploinsufficiency in cardiac progenitor cells, and is responsible for the proper expression of Nkx2.5 in adult cardiac side population stem cells. These effects are consistent with the temporary presence of desmin in the nuclei of differentiating cardiac progenitor cells and its physical interaction with transcription factor complexes bound to the enhancer and promoter elements of the *nkx2.5* gene. These findings introduce desmin as a newly discovered and unexpected player in the regulatory network guiding cardiomyogenesis in cardiac stem cells.

## Abstract (precis)

**Summary:** These data may be the first evidence for a nuclear function of an intermediate filament protein. In cardiac stem cells, desmin interacts with transcription factor complexes influencing Nkx 2.5 transcription.

## INTRODUCTION

The heart is the first organ developing in mammalian embryos and has to be functional throughout life (Vincent and Buckingham, 2010). Cardiomyogenesis is guided by a hierarchically structured network of transcription factor (TF) genes receiving versatile extrinsic and intrinsic signals during commitment of primitive mesoderm to the cardiogenic lineage and differentiation of cardiac progenitor cells (CPCs) (Taubenschmid and Weitzer, 2012). The transition from primitive mesoderm to early stages of CPCs is facilitated by the TFs brachyury (King et al., 1998), eomesodermin (Costello et al., 2011), and Mesp1 (Bondue and Blanpain, 2010; Bondue et al., 2008; David et al., 2008, 2011) and certainly by many others that have not been well studied in this respect yet. Nkx2.5 and GATA4 are expressed downstream of the above mentioned TFs, and contribute together with Isl1 to fate decisions along the myocardial lineage (Den Hartogh et al., 2015; Dorn et al., 2015), finally leading to the formation of the four-chambered mammalian heart (Srivastava, 2006).

The homeobox TF Csx/Nkx2.5, a homolog of the invertebrate's TF tinman (Akazawa and Komuro, 2005; Chen and Schwartz, 1995; Harvey et al., 2002; Schwartz and Olson, 1999), plays a critical role in ensuring spatial and temporal discrimination between progenitor and differentiated states during cardiomyogenesis (Dorn et al., 2015; Prall et al., 2007), and contributes to homeostasis in the adult heart (Reamon-Buettner and Borlak, 2010). Nkx2.5 expression is regulated by a vast number of TFs, such as Mesp1, Tbx5, and GATA4 (Taubenschmid and Weitzer, 2012). On the other hand, Nkx2.5 interacts with TFs such as GATA4, Tbx5, and Mef2C in regulating downstream target genes. Cardiomyogenesis is severely hampered when Nkx2.5 is mutated or absent in vertebrates (Bodmer, 1993; Bruneau et al., 2000; Chen and Fishman, 1996; Kasahara et al., 2000), and mutant Nkx2.5 cause severe congenital heart diseases in humans (Reamon-Buettner and Borlak, 2010). The consequences of *nkx2.5* haploinsufficiency in mice (Biben et al., 2000; Jay et al., 2005) and a negative auto-regulatory Nkx2.5 feedback loop (Prall et al., 2007; Tanaka et al., 1999) suggest that fine-tuning of the Nkx2.5 expression level is critical for proper CPC specification, cardiomyogenesis, and homeostasis of the adult heart (Akazawa and Komuro, 2003). This hypothesis is strengthened by the observation that over-expression of the muscle-specific intermediate filament (IF) protein desmin causes an up-regulation of brachyury and Nkx2.5 expression in CPCs followed by a significantly improved cardiomyogenic differentiation in embryoid bodies (EBs) (Hofner et al., 2007).

Desmin is one of the earliest cardiac muscle specific proteins expressed in mesodermal cells committed to the myocardial lineage (Capetanaki et al., 2015; Kuisk et al., 1996; Li and Capetanaki, 1994), in satellite cells (Allen et al., 1991), and in cardiac muscle side population stem cells (CSPCs) (Pfister et al., 2005). Desmin is a type III IF protein and a member of a large family of more than 70 proteins (Oshima, 2007). These proteins were originally believed to provide a static framework supporting the cytoarchitecture of all metazoan cells but there is accumulating evidence demonstrating that IFs are highly dynamic structures and that their subunits seem to contribute to a plethora of regulatory processes involved in differentiation, homeostasis, aging, and disease (Hyder et al., 2011). Knock out of the *desmin* gene, although without any obvious phenotypic consequences during murine embryogenesis (Li et al., 1996; Milner et al., 1996), causes severe cardiac defects during adulthood (Mavroidis et al., 2015; Milner et al., 2000, 1996; Psarras et al., 2012; Thornell et al., 1997). In line with these data, a variety of mutations in the *desmin* gene have been linked to human skeletal and cardiac myopathies (Capetanaki et al., 2015). Absence of desmin in muscle cells leads to structural and functional mitochondrial defects (Milner et al., 2000; Papathanasiou et al., 2015; Weisleder et al., 2004), however, the consequences of its deficiency to nuclear functions is the most intriguing one. Skeletal muscle specific myogenic TFs MyoD and myogenin are down-regulated in the absence of desmin in C2C12 myoblasts (Li et al., 1994) and during embryonic stem cell (ESC) differentiation in *desmin* knockout EBs (Weitzer et al., 1995). Further, overexpression of desmin in differentiating ESCs increases the expression of the TFs brachyury and Nkx2.5 in developing CPCs (Hofner et al., 2007), and accelerates the commitment and differentiation of primitive mesodermal cells to rhythmically contracting cardiomyocytes (CMCs). Deletion of desmin's amino-terminal domain or mutation of serine residues 6, 7, and 8 or 31 and 32 to alanine, causes a significant down regulation of early Nkx2.5 expression in EBs and severely hampers cardiomyogenesis (Höllrigl et al., 2007, 2002). Finally, *in vivo*, desmin and Nkx2.5 co-localize and are first detectable in the precardiac mesoderm between embryonic day 7.5 and 7.8 (Kasahara et al., 1998; Kuisk et al., 1996).

Abbreviations:

CSPC: cardiac side population stem cell
CPC: cardiac progenitor cell
MCE: cardiac specific enhancer region
CMC: cardiomyocyte
CVPC: cardiovascular progenitor cell
ChIP: chromatin immuno-precipitation
EB: embryoid body
ESC: embryonic stem cell
FBS: fetal bovine serum
IF: intermediate filament
LUC: luciferase
PE: proximal enhancer
PEPR: proximal enhancer and promoter region
shRNA: short hairpin RNA
TF: transcription factor

All these results suggest that desmin may also influence transcription of genes important for early cardiomyogenesis, in particular Nkx2.5, directly. This notion is supported by data demonstrating that desmin and vimentin interact with DNA *in vitro* (Tolstonog et al., 2005; Traub and Shoeman, 1994b). Desmin has been detected in nuclei of BHK21 cells (Kamei, 1986) and nestin in nuclei of brain tumor cells (Krupkova et al., 2011). It has been suggested that vimentin enters the nucleus via a piggyback mechanism (Hartig et al., 1998) and binds to DNA via its amino-terminal domain particularly at the nuclear matrix attachment regions (Tolstonog et al., 2001). Strong nuclear vimentin signals have also been found in lymph node metastasis from nasopharyngeal carcinoma (Luo et al., 2012). These independent lines of evidence suggest that type III IF proteins may enter the nucleus under certain circumstances, and influence transcriptional processes by interacting with DNA and proteinaceous components of the chromatin directly.

Identification of genes acting upstream of *nkx2.5* is crucial for understanding the transcriptional network and the interwoven paracrine and autocrine signals that regulate the development of primitive mesoderm to functional CMCs. Thus, we decided to test if desmin promotes cardiogenic commitment and myocardial differentiation directly by influencing the transcriptional network in CPCs. Herein, we demonstrate that desmin is indeed involved in a temporary, cell autonomous molecular process influencing Nkx2.5 transcription in CPCs during early cardiomyogenesis and in adult CSPCs. Desmin activates transcription of *nkx2.5* reporter genes and rescues the haploinsufficient phenotype in heterozygous *nkx2.5* knockout EBs by increasing Nkx2.5 expression. Moreover, absence of desmin in CSPCs from adult *desmin*-knockout mice causes a reduced Nkx2.5 expression. These effects are mediated by the temporary presence of desmin in the nuclei of differentiating CPCs and the physical interaction of desmin with enhancer and promoter elements of the *nkx2.5* gene. Thus, desmin contributes to the transcriptional regulation of the *nkx2.5* gene in CPCs during a short period of time at the beginning of cardiomyogenesis and in CSPCs in the hearts of adult mice.

## RESULTS

### Desmin influences expression of the *nkx2.5* gene in a proximal promoter and minimal cardiac specific enhancer dependent manner

**Fig. 1.**
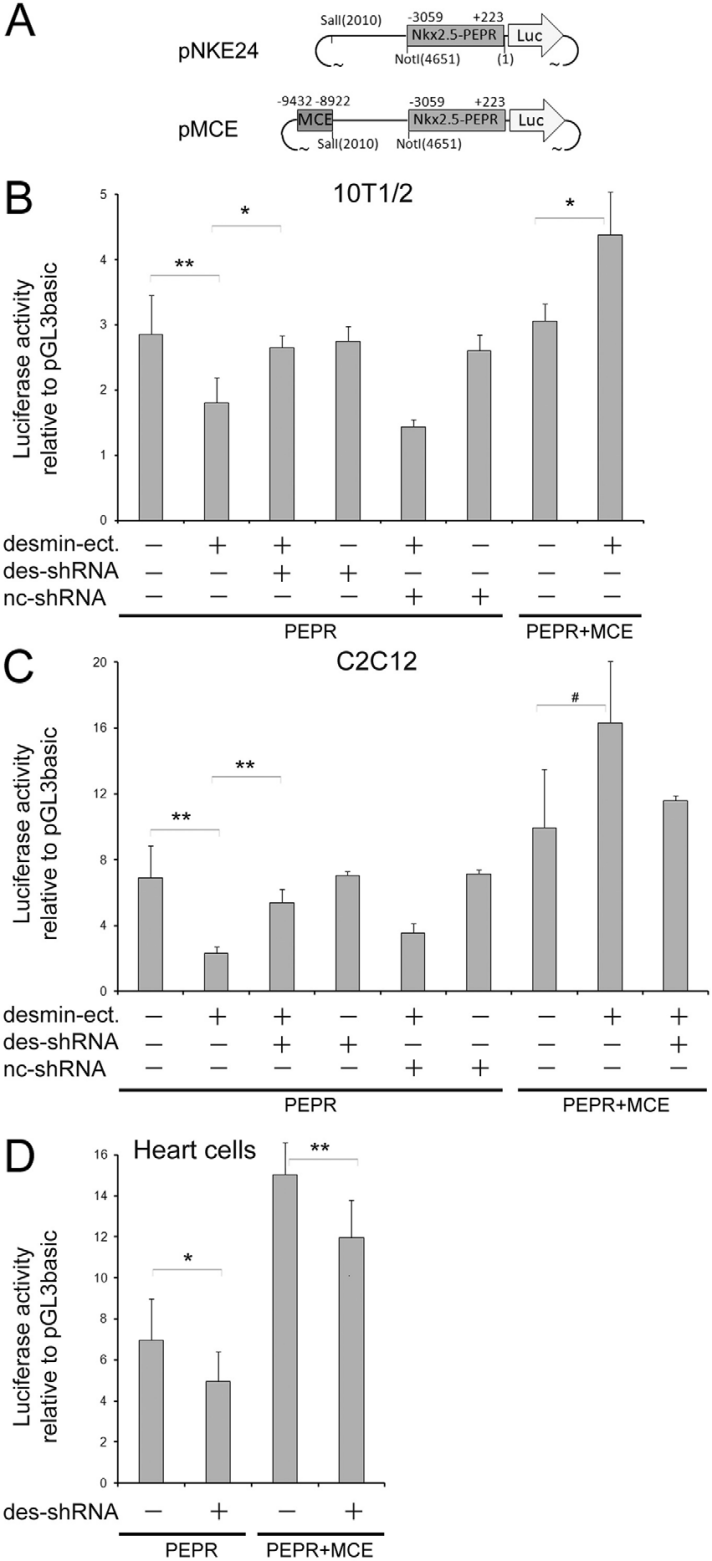
**The proximal promoter and the minimal cardiac-specific enhancer confer desmin-regulated Nkx2.5 expression.** (A) Maps of plasmids used for the transient transfection assays. Numbers on top of the scheme depict the position of the regions of the 5′UTR of the *nkx2.5* gene inserted into the pGL3b plasmid. Numbers in brackets below the scheme depict the positions of the *Sal*I and *Not*I sites of pGL3b were the MCE and PEPR were inserted. Reporter plasmids were transfected together with a plasmid ectopically expressing desmin under the control of the RSV promoter (desmin-ect.), a shRNA knockdown plasmid, interfering with desmin expression (des-shRNA), and a negative control shRNA plasmid (nc-shRNA), not interfering with *desmin* mRNA expression. LUC activity of reporter plasmids were normalized to the activity measured in the presence of the promoter-less pGL3b plasmid and to transfection efficiency, using a *Renilla* LUC reporter plasmid. (B) PEPR- and MCE-driven LUC activity in 10T1/2 fibroblasts expressing minimal amounts of endogenous desmin. (C) PEPR- and MCE-driven LUC activity in C2C12 myoblasts expressing significant levels of endogenous desmin and skeletal muscle-specific TFs. (D) PEPR- and MCE-driven LUC activity in primary neonatal heart cells expressing significant levels of endogenous desmin and cardiac-specific TFs. Data are presented as mean±s.d. Data shown in B,C are from five independent experiments with the exemption for the right outermost bar in C where *n*=2; D, *n*=2. *P<0.05, **P<0.01, #*P*=0.056.

Previous results have demonstrated that early and increased expression of desmin in differentiating embryonic stem cells (ESCs) promotes cardiomyogenesis (Hofner et al., 2007). This becomes evident by temporarily increased expression of the cardiac transcription factor (TF) gene *nkx2.5,* followed by an increase in the number of rhythmically contracting cardiomyocytes (CMCs) in embryoid bodies (EBs). To test whether desmin influences Nkx2.5 expression in a cell-autonomous fashion or not, we chose two cell lines with different myogenic potentials and the ability to activate the *nkx2.5* gene. The first cell line, 10T1/2 fibroblasts, expresses small amounts of desmin, if any at all, but have a latent myogenic (Weintraub et al., 1989) and cardiogenic (Zhou et al., 2012) potential, whereas the second one, C2C12 myoblasts, expresses significant amounts of desmin and can be induced to differentiate into multinucleated myotubes (Yaffe and Saxel, 1977). The influence of desmin on *nkx2.5* gene expression was monitored first with the luciferase (LUC) reporter plasmid pNKE24 (Searcy et al., 1998), containing the proximal enhancer and promoter region (PEPR) of the *nkx2.5* gene and then with the LUC reporter plasmid pMCE, containing the minimal cardiac specific enhancer region (MCE) (Lien et al., 1999) in addition to the PEPR (Fig. 1A; for precise localization along the *nkx2.5* gene see Fig. 4A).

Co-transfection of 10T1/2 fibroblasts with pNKE24 and a desmin-expressing plasmid (desmin-ect.) resulted in a significant decrease of the LUC activity (Fig. 1B). This inhibitory effect of desmin could be reverted by co-transfection with a desmin-specific short hairpin RNA (shRNA) plasmid (des-shRNA) resulting in desmin expression reduced to basal levels (Fig. S1). In sharp contrast, co-transfection with pMCE conferred a desmin-dependent increase in the expression of the *nkx2.5* LUC reporter.

Transfection of C2C12 myoblasts with pNKE24 resulted in a much larger basal LUC activity compared to 10T1/2 fibroblasts, suggesting that C2C12 myoblasts express a more appropriate set of transcriptional co-activators of the *nkx2.5* gene than 10T1/2 cells (Fig. 1C). Nonetheless, again co-expression of desmin resulted in an attenuation of the *nkx2.5* LUC reporter expression when only the PEPR was present in pNKE24, while des-shRNA expression partially compensated the negative effect caused by desmin. Addition of the MCE to the PEPR resulted in significantly increased pMCE-mediated LUC activity and desmin co-expression caused an additional 40% increase of the LUC activity which was attenuated by des-shRNA expression. These two sets of concordant data suggest that desmin influences Nkx2.5 expression in fibroblasts and myoblasts through the MCE and the PEPR of the *nkx2.5* gene.

To test if the effect of desmin on Nkx2.5 expression has some physiological relevance in maturing CMCs, which already express substantial amounts of desmin, we co-transfected primary heart cells from newborn mice with the LUC reporter plasmids pNKE24 and pMCE and the des-shRNA plasmid (Fig. 1D). Transfection with the PEPR containing reporter plasmid pNKE24 led to a two-fold increase in LUC activity as compared to 10T1/2 fibroblasts, and co-transfection with the des-shRNA plasmid reduced the LUC activity to 60% of the basal activity. Likewise, transfection of heart cells with the MCE+PEPR containing reporter plasmid pMCE resulted in a five-fold increase in LUC activity, which again was reduced to 72% by desmin-specific des-shRNA. The positive effect of desmin on PERP activity in primary cardiac cells, in contrast to the negative effect in 10T1/2 and C2C12 cells, suggests that desmin requires a cardiac cell specific set of co-factors to positively influence the PEPR region of the *nkx2.5* gene, whereas presence of the MCE conferred activation in all tested cell types. These results together indicate that desmin contributes to the modulation of Nkx2.5 expression in developing muscle cells and substantiate previous findings that desmin over-expression in ESCs undergoing cardiomyogenesis increases Nkx2.5 expression.

### Expression of desmin rescues early cardiomyogenesis in *nkx2.5* haploinsufficient cardiac progenitor cells

**Fig. 2.**
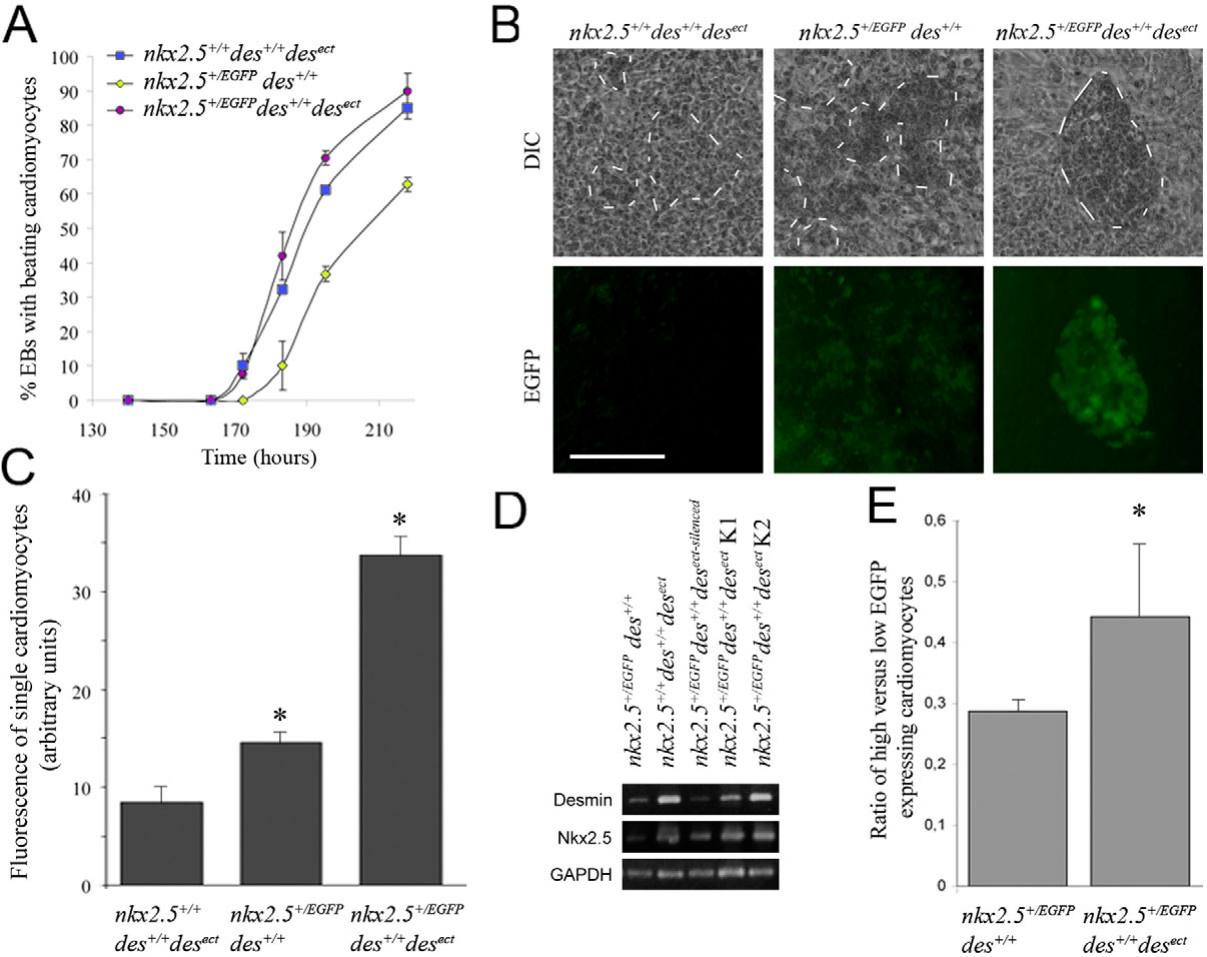
**Desmin expression rescues cardiomyogenesis in haploinsufficient *nkx2.5::EGFP* cardiac progenitor cells by upregulation of Nkx2.5 expression.** (A) Cardiomyogenesis in EBs made from ESCs over-expressing desmin (*nkx2.5^+/+^ des^+/+^ des^ect^*)*,* in Nkx2.5-haploinsufficient EBs with a heterozygous *nkx2.5::EGFP* knock-in allele (*nkx2.5^+/EGFP^ des^+/+^*)*,* and in EBs from ESCs over-expressing desmin in the presence of the *nkx2.5^EGFP^* allele (*nkx2.5^+/EGFP^ des^+/+^des^ect^*). (B) Expression of the *nkx2.5^EGFP^* allele in rhythmically contracting CMCs in EBs at day 10. Differential interference contrast (DIC) was used to locate rhythmically contracting CMCs (curtailed by dashed white lines) and corresponding fluorescence images (EGFP). Scale bar: 100 µm. (C) Intensity of the EGFP fluorescence signal in single living CPCs analyzed by fluorescence microscopy and image analysis. Control, background fluorescence in *nkx2.5^+/+^ des^+/+^ des^ect^* CPCs. Number of cells *n*=267. *P<0.05. (D) RT-PCR analysis of the expression levels of *nkx2.5* mRNA from the wild-type allele in 7-day old EBs with genotypes as shown in A and a clonal cell line with a coincidentally silenced *des^ect^* transgene. K1 and K2, two different clonal cell lines with the *nkx2.5^+/EGFP^ des^+/+^des^ect^* genotype. GAPDH, loading control. (E) Flow cytometry analysis of EGFP^high^ versus EGFP^low^ expression in CPCs from haploinsufficient *nkx2.5^+/EGFP^ des^+/+^* and rescued *nkx2.5^+/EGFP^ des^+/+^des^ect^* EBs 180 h post-aggregation. Data are presented as mean±s.d., *n*=3; *P<0.05.

To demonstrate that desmin promotes expression of the endogenous *nkx2.5* locus in ESC-derived cardiac progenitor cells (CPCs) and differentiating CMCs, we generated heterozygous *nkx2.5^+/EGFP^* ESCs with one allele of the *nkx2.5* gene converted to an *EGFP* reporter allele by homologous recombination with a *nkx2.5::EGFP* knock-in vector (Hidaka et al., 2003). Heterozygosity at the *nkx2.5* locus causes haploinsufficiency (Biben et al., 2000; Kasahara et al., 2000) with evident functional consequences at the very beginning of cardiomyogenesis in EBs (Fig. 2A). In EBs derived from *nkx2.5^+/EGFP^ des^+/+^* ESCs the onset of cardiomyogenic differentiation was significantly delayed and reduced when compared to wild type *nkx2.5^+/+^des^+/+^* EBs (Weitzer et al., 1995) and desmin over-expressing *nkx2.5^+/+^des^+/+^des^ect^* EBs (Hofner et al., 2007). By contrast, desmin over-expression in *nkx2.5^+/EGFP^ des^+/+^des^ect^* EBs reversed the nkx2.5-related haploinsufficency phenotype and cardiomyogenesis commenced as in *nkx2.5^+/+^des^+/+^des^ect^* EBs (Fig. 2A). Fluorescence microscopy revealed a strongly increased EGFP fluorescence signal in the presence of desmin in differentiating *nkx2.5^+/EGFP^ des^+/+^des^ect^* CMCs (Fig. 2B). Accordingly, quantification of EGFP intensity in single contracting CMCs by image analysis of fluorescence microscopy data demonstrated increased fluorescence intensity, and therefore increased expression of the *nkx2.5^EGFP^* allele in CMCs (Fig. 2C). The effect of desmin on the *nkx2.5^+^* allele was also demonstrated by RT-PCR using *desmin*- and *nkx2.5*-specific primer pairs (Fig. 2D). Likewise, quantification of EGFP fluorescence in CPCs and early CMCs from EBs at day 7.5 using flow cytometry provided evidence for an increased number of cells with a strongly increased EGFP fluorescence in the presence of desmin (Fig. 2D), suggesting that desmin enhances even mono-allelic Nkx2.5 expression in differentiating CMCs, and thus rescues this Nkx2.5-related haploinsufficient phenotype.

### Absence of desmin expression in cardiac side population stem cells from adult *desmin^−/−^* mouse hearts causes reduced Nkx2.5 expression

**Fig. 3.**
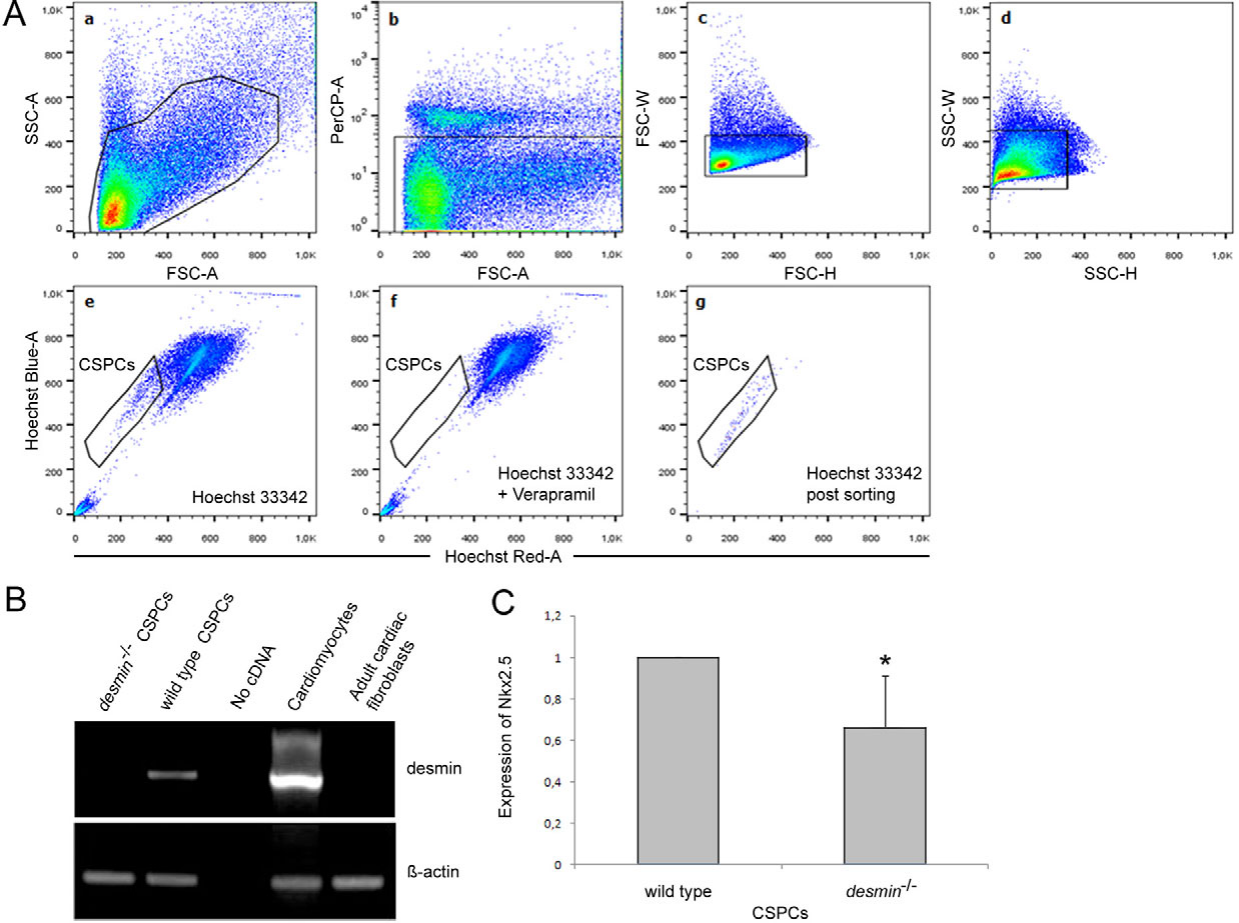
**Absence of desmin expression in cardiac side population stem cells from adult *desmin^−/−^* mice causes reduced Nkx2.5 expression.** (A) Gating strategy for the isolation of CSPCs from 90-day old mouse hearts. (a) Total mononucleated CMC-depleted cells from wild-type and *desmin^−/−^* mice were stained with Hoechst 33342 and 7-Aminoactinomysin D and were gated according to cell size [forward scatter (FSC-A)] and granularity [side scatter (SSC-A)]. (b) 7-Aminoactinomysin D was used to exclude the dead cells from the analysis. (c,d) Two additional gatings for cell size (FSC-W/FSC-H) and granularity (SSC-W/SSC-H) were performed for doublet discrimination. (e) Hoechst 33342 staining was used to identify and to gate the Hoechst Blue^low^/Hoechst Red^low^ CSPCs. (f) Verapamil was used to block the ABC-transporter dependent Hoechst 33342 efflux and to confirm the gate for preparative FACS of CSPCs. (g) CSPCs population after sorting in the presence of Hoechst 33342. (B) RT-PCR analysis of the expression levels of *desmin* mRNA in wild-type and *desmin^−/−^* CSPCs maintained in culture for 10 days. (C) Quantitative RT-PCR analysis of the expression levels of *nkx2.5* mRNA in wild-type and *desmin^−/−^* CSPCs. Data are presented as mean±s.d., *n*=5; *P<0.05.

To demonstrate the *in vivo* relevance of desmin in sustaining proper Nkx2.5 expression levels in adult cardiac stem cells, we isolated cardiac side population stem cells (CSPCs) from 90 day old *desmin^−/−^* mice (Fig. 3A) and compared the expression levels of *nkx2.5* by qRT-PCR to desmin-expressing CSPCs isolated from wild type mice of the same age. First we verified *desmin* expression in CSPCs (Fig. 3B), as previously described (Yamahara et al., 2008). In the absence of any desmin expression, *nkx2.5* levels were reduced by one third in CSPCs from adult mouse hearts (Fig. 3C), indicating that desmin partially contributes to the maintenance of Nkx2.5 expression in adult CSPCs *in vivo* as well.

### Desmin is a component of transcription factor complexes interacting with enhancers of the *nkx2.5* gene at the beginning of cardiomyogenesis

**Fig. 4.**
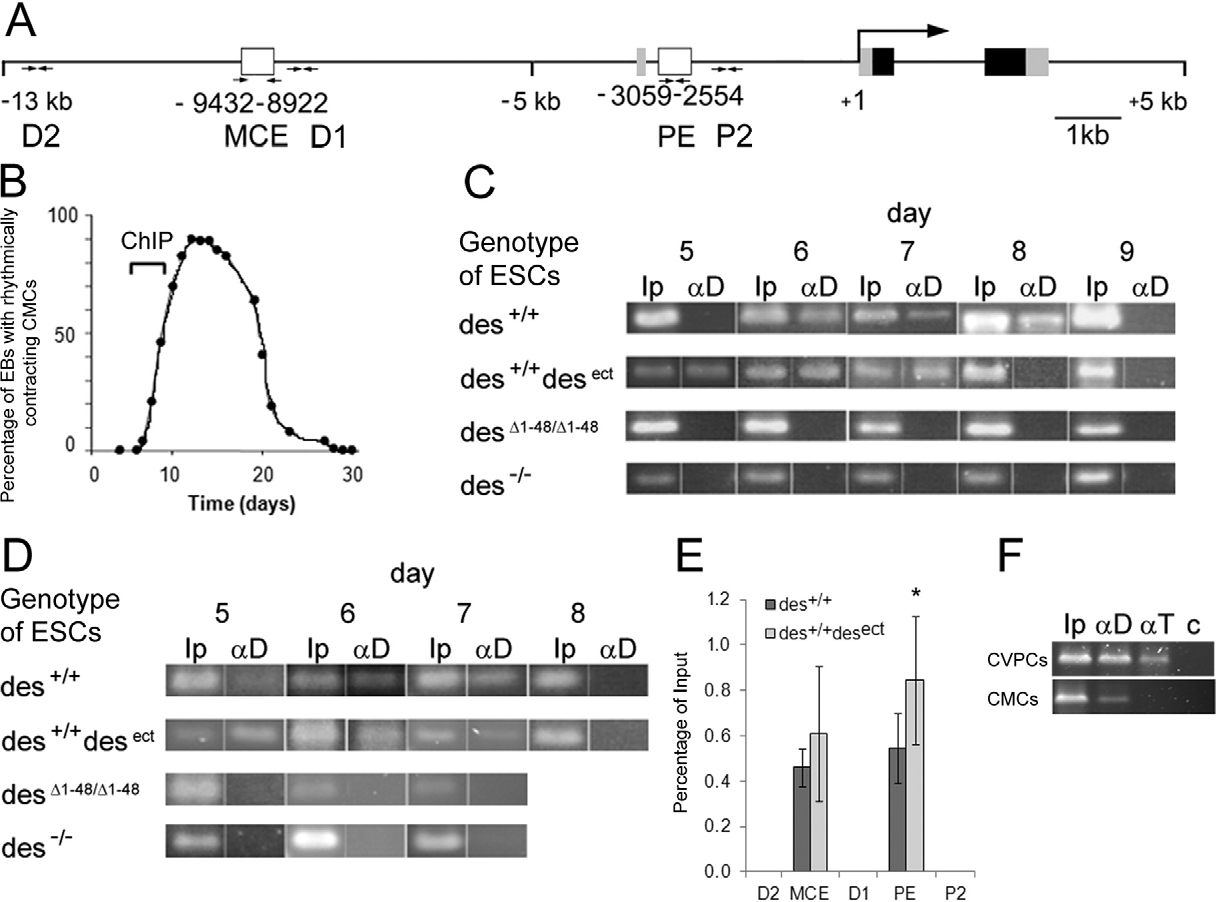
**Desmin is a component of transcription factor complexes interacting with regulatory regions of the *nkx2.5* gene at the beginning of cardiomyogenesis.** (A) Map of the *nkx2.5* gene located in negative orientation on mouse chromosome 17 (NC_000083.6; base pair −1 corresponds to position 26.841.565 and A of ATG to 26.841.355 on chromosome 17). The minimal cardiac specific enhancer (MCE) and the proximal enhancer (PE) are indicated as white boxes. Numbers of base pairs at the beginning and end indicate distances relating to the transcription start site. Gray-black boxes, exons; black parts, open reading frame. Pairs of arrows indicate primer binding sites used to amplify DNA obtained by ChIP with desmin specific antibodies. (B) Development of rhythmically contracting CMCs in EBs over time. Bracket indicates the time frame when samples of cells were picked for ChIP analysis at the beginning of cardiomyogenesis. (C) ChIP with desmin-specific antibodies and a PCR primer pair specific for the PE of the *nkx2.5* gene in *des^+/+^, des^+/+^des^ect^*, *des^Δ1-48/Δ1-48^*, and *des^−/−^* EBs between days 5 and 9 after aggregation. Ip, input DNA before pull-down with antibodies; αD, ChIP with desmin specific antibodies. Genotypes of ESC lines are indicated on the left. Representative PCR data out of five independent biological samples. (D) Representative PCR analysis of same ChIP samples used in C with primer pairs specific for the MCE from days 5 to 8. (E) Mean intensity of the PCR signals (αD) as percentage of the input signal (Ip) from five independent biological samples at regions D2 to P2 along the 5′UTR of the *nkx2.5* gene. Error bars, s.d.; *P<0.05. (F) ChIP in differentiating CVPCs and in primary heart cells from newborn mice (CMCs) with desmin-specific antibodies (αD), brachyury-specific antibodies (αT), and IgG (c), as negative control with a PE-specific PCR primer pair.

To further delineate the cell-autonomous effect of desmin on Nkx2.5 expression in CPCs, we used chromatin immuno-precipitation (ChIP) to screen the *nkx2.5* gene for the presence of desmin in TF complexes bound to DNA. Primer pairs for PCR were chosen to amplify the minimal cardiac specific enhancer (MCE; also called AR1), located between base pairs −9432 and −8922 of the murine *nkx2.5* gene (Brown et al., 2004; Lien et al., 1999) and the essential proximal enhancer (PE; also called AR2) (Brown et al., 2004; Searcy et al., 1998), located between base pairs −3059 and −2554, and adjacent 5′- and 3′-flanking sequences (Fig. 4A). As a source of CPCs we used EBs expressing wild-type and mutant desmin alleles. ChIP with desmin-specific antibodies was performed at the time when first rhythmically contracting CMCs are observed in EBs (Fig. 4B). At that time desmin is exclusively expressed in CPCs and early CMCs (Hofner et al., 2007; Weitzer et al., 1995). Desmin interacted with the PE but not with adjacent DNA sequences in CPCs from wild type (*des^+/+^*) EBs between day 6 and 8 (Fig. 4C). In desmin over-expressing EBs with one additional RSV-driven desmin allele (*des^+/+^des^ect^*), association of desmin with the *nkx2.5* gene was already observed from day 5 on but did not last longer than in *des^+/+^* EBs. In EBs, homozygously expressing a mutant desmin lacking the amino-terminus (*des^Δ1-48/Δ1-48^*), as in desmin knock-out EBs (*des^−/−^*), no signals could be detected, suggesting that these 48 amino acids of the amino-terminus potentially mediate this interaction of desmin with DNA or TFs, or might be necessary for nuclear transport. Likewise, desmin was present in a TF complex interacting with the MCE of the *nkx2.5* gene in *des^+/+^* and *des^+/+^des^ect^* CPCs between days 5 and 7, but not in *des^Δ1-48/Δ1-48^* and *des^−/−^* CPCs (Fig. 4D). Data from five independent biological replicates demonstrated a desmin concentration dependent increase in the number of desmin containing TF complexes bound to the PE and MCE of the *nkx2.5* gene in ESC-derived CPCs (Fig. 4E). As a positive control, we repeated these experiments with a Mesp1 specific antibody and confirmed the already reported interaction of Mesp1 with the MCE and PE of the *nkx2.5* gene (Bondue et al., 2008). Desmin was also present in a TF complex bound to the PE in differentiating cardiovascular progenitor cells (CVPCs), isolated as phenotypically stable cell lines from hearts of newborn mice (Hoebaus et al., 2013), and in primary heart cells from newborn mice (Fig. 4F). Additionally, desmin could be identified as a component of TF complexes interacting with the *nanog*, *brachyury*, *mesp1*, and *desmin* genes in ESC- and CVPC-derived CPCs (Table S2), suggesting that nuclear localization of desmin during early cardiomyogenesis might even contribute to the transcriptional regulation of several genes.

### Desmin can be localized in nuclei of differentiating cardiac progenitor cells and immature cardiomyocytes

**Fig. 5.**
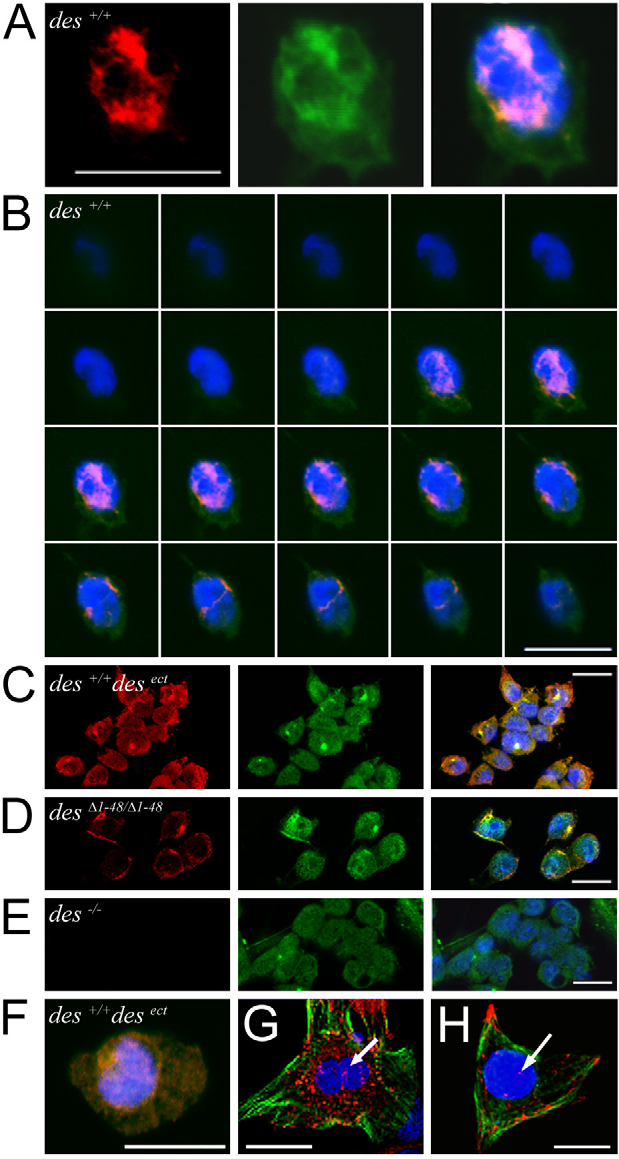
**Localization of desmin in nuclei of differentiating cardiac progenitor cells and premature cardiomyocytes.** Confocal immunofluorescence micrographs of ESC-derived CPCs at different developmental stages (A-F) and primary premature CMCs (G,H). Desmin (red, A-H), vimentin (green, A-F), and cardiac troponin T (green, G,H) were recognized by specific antibodies. Right images in A,C-E, and images B,F-H are merged images. DNA was counterstained with DAPI (blue). Genotypes of ESC-derived CPCs as indicated. (A) Nuclear localization of desmin in a differentiating ESC-derived CPC at day 5.5. The image is taken from the middle of 20 *Z*-stack optical sections through the nucleus. (B) 20 *Z*-stack optical sections of cell shown in A (Step size: 0.5 µm). (C) Undifferentiated *des^+/+^des^ect^* ESCs. (D) Differentiating *des^Δ1-48/Δ1-48^* ESCs. (E) Differentiating *des^−/−^* ESCs. (F) Single *des^+/+^des^ect^* ESCs with desmin and vimentin positive nucleus. (G,H) Primary immature CMCs from newborn mice. Images 6 out of 12 image stacks. Arrows, intra-nuclear desmin spots. Scale bars: 18 µm.

Identification of desmin-containing TF complexes bound to the *nkx2.5*, *nanog*, *brachyury*, *mesp1*, and *desmin* genes and the premature but not permanent up-regulation of Nkx2.5 expression in *des^+/+^des^ect^* ESC-derived CPCs (Hofner et al., 2007) suggest that desmin might be present in nuclei of CPCs only during a relative short period of time, and therefore had been overlooked in former studies. Hence we screened different developmental states of CPCs derived from ESC and CVPC lines stained with desmin specific antibodies, by optical sectioning of nuclei with a confocal immunofluorescence microscope, and determined the percentage of cells with an unambiguous presence of desmin in the nucleus (Table 1). In developing ESC-derived CPCs, desmin protein could be detected in many cells at day 5.5 after aggregation, which is two days after *desmin* mRNA has become detectable by northern blotting (Höllrigl et al., 2002) and RT-PCR (Hofner et al., 2007). Indeed, in about 7% of these cells desmin was found in the nucleus (Fig. 5A,B; Table 1), partially co-localizing with vimentin. As expected, desmin was not detected in undifferentiated *des^+/+^* ESCs (Fig. S2A) which, however, express low amounts of vimentin, consistent with previous data in human ESCs (Van Hoof et al., 2008). Ectopic expression of desmin in *des^+/+^des^ect^* ESCs resulted in the cytoplasmic localization of desmin in all undifferentiated ESCs (Fig. 5C) and in 0.2% of these cells desmin was detected in the nucleus (Fig. 5F). These few cells are most likely already prematurely differentiating along the myocardial lineage, because over-expression of desmin favours myocardial commitment of ESCs (Hofner et al., 2007). In contrast to wild type desmin, mutant desmin protein lacking the amino-terminus was never localized in the nuclei of *des^ Δ1-48/Δ1-48^* ESCs (Fig. 5D). Any potentially misleading cross-reactivity of the desmin antibodies were excluded by the absence of a detectable signal in differentiating *des^−/−^* ESCs (Fig. 5E). In undifferentiated CVPCs nearly no desmin was detected by immunofluorescence microscopy (Table 1 and Fig. S2C), whereas at day 7 after aggregation and initiation of myocardial differentiation desmin localized to the nucleus in 10% of the CVPC-derived CPCs (Table 1; Fig. S2D,E). This percentage decreased to 4% at day 13 when CMCs, already contracting from day 11, became even more mature. However, a small number of the emerging cardiac troponin T-positive CMCs still had some desmin localized in the nucleus (Fig. S2F,G).

**Table 1.**
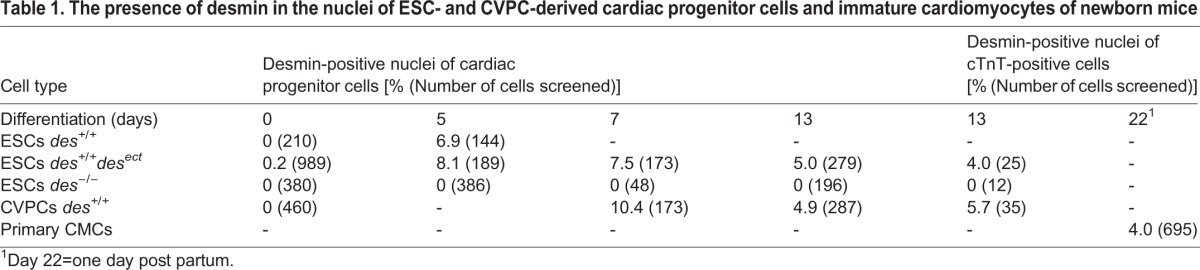
**The presence of desmin in the nuclei of ESC- and CVPC-derived cardiac progenitor cells and immature cardiomyocytes of newborn mice**

In hearts from neonatal mice, desmin was present in 4% of the nuclei of primary immature CMCs (Table 1, Fig. 5G,H; Fig. S3A). By contrast, desmin was neither found in nuclei of desmin-positive cardiac fibroblast-like cells (Fig. S3B), nor in nuclei of fully differentiated CMCs (Fig. S3C), wherein desmin was predominantly localized at the Z-discs of myofibrils. Taken together, these data demonstrate that desmin was exclusively found in nuclei of CPCs which were committed to the myocardial lineage but had not fully completed differentiation to CMCs.

### mCherry-tagged desmin proteins are visualized in the nucleus of cardiac progenitor cells

**Fig. 6.**
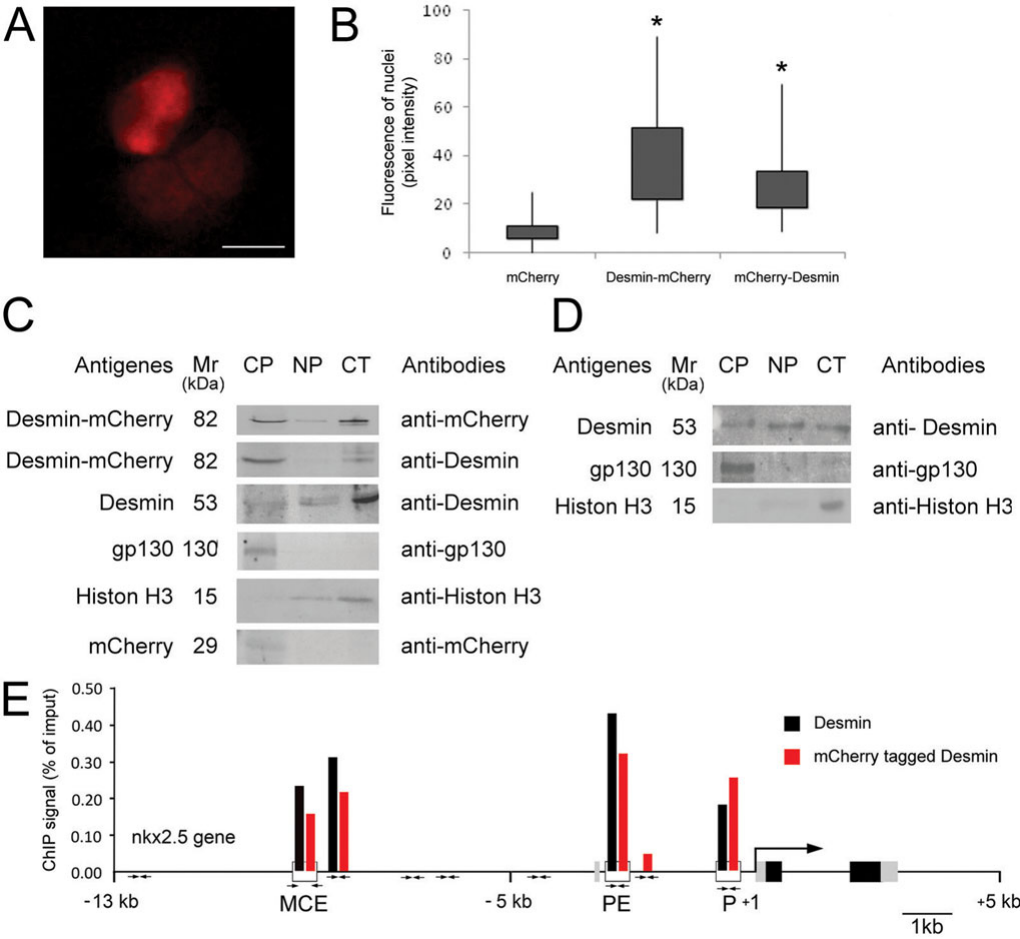
**Desmin and mCherry-tagged desmin partially co-segregate with chromatin in cardiac progenitor cells and primary heart cells and interact with the *nkx2.5* gene.** (A) Localization of mCherry-desmin in the nucleus. Fluorescence microscopy image of isolated nuclei from mCherry-desmin-expressing ESC-derived CPCs at day 6.7 after aggregation. Scale bar: 3 µm. (B) Quantification of desmin fusion proteins in the nuclei of CPCs. CVPC lines expressing only mCherry, or desmin-mCherry and mCherry-desmin, respectively, were differentiated for 9.3 days. Box plot, median ±25% of data. Whiskers, minimal and maximal values, respectively. Data are from four independent experiments. Numbers of nuclei: mCherry, *n*=352; Desmin-mCherry, *n*=360; mCherry-Desmin, *n*=406. *P<0.0001. (C) Desmin-mCherry and desmin associate with the chromatin fraction. Western blot analysis of cytoplasmic (CP), nucleoplasmic (NP) and chromatin (CT) fractions from desmin-mCherry (rows 1-5) and mCherry only (row 6) expressing CVPC-derived CPCs with antibodies specific for mCherry, desmin, the transmembrane protein gp130, and histone H3. Notably, due to nuclear preparation constraints, the CP fraction contained 1/20 the amount of proteins loaded in the NP and CT fractions. (D) Desmin partially associates with the chromatin fraction in primary immature heart cells from 3-days postpartum mouse hearts. Fractionation of cells and analysis as in C. (E) ChIP analyses with mCherry-specific antibodies in differentiating, mCherry-tagged, desmin-expressing CPCs derived from ESC and CVPC lines (red bars). ChIP signal intensity is depicted as bars on top of the *nkx2.5* map highlighting the minimal cardiac specific enhancer (MCE), the proximal enhancer (PE) and the promoter (P), and compared to ChIP data obtained with desmin specific antibodies (black bars) in wild-type cell lines. Pairs of small arrows indicate primer pairs used for PCR analysis. Data are from two independent experiments.

To further confirm the nuclear localization of desmin without using desmin-specific antibodies, we generated ESC and CVPC lines, expressing either a desmin-mCherry or a mCherry-desmin fusion protein, and as controls, expressing merely mCherry. In fibroblasts these fusion proteins integrated well in the pre-existing vimentin IF network but displayed a diffuse and spotty staining in the cytoplasm of undifferentiated ESCs and CVPCs (Fig. S4A) which express vimentin and cytokeratins but most likely do not contain IFs (de Souza Martins et al., 2011; Gao et al., 1994). Although expressed under the control of the CMV promoter in all cells, mCherry-tagged desmin was found in nuclei of less than 10% of the differentiating CVPCs (Fig. S4B). Nuclear localization of the mCherry-tagged desmin proteins was demonstrated in nuclei isolated and purified from differentiating CPCs (Fig. 6A; Fig. S4C). Again, only 10.7% (*n*=440) of the purified nuclei of CVPC-derived CPCs contained the mCherry-tagged desmin proteins. Quantification of the nuclear fluorescence signals in differentiating CPCs demonstrated that, similar to endogenous desmin, both desmin-mCherry and mCherry-desmin fusion proteins, but not mCherry alone, can enter the nucleus (Fig. 6B).

Analysis of the cytoplasmic, nucleoplasmic, and chromatin fractions of mCherry-tagged desmin expressing CPCs by western blot analysis with mCherry specific antibodies corroborated the association of the mCherry-tagged desmin proteins with chromatin (Fig. 6C; Fig. S4D). mCherry expressed in control cell lines was exclusively found in the cytoplasmic fraction. As controls, the same cellular fractions were tested with desmin antibodies and the nuclear localization of endogenous desmin was confirmed. Furthermore, use of gp130 and histone H3 specific antibodies, respectively, demonstrated the efficient separation of cytoplasm and membranes from the chromatin. Finally, fluorescence microscopy of isolated nuclei confirmed the absence of any IFs at the nuclear periphery.

Applying the same isolation procedure to murine heart cells, isolated three days post-partum, also demonstrated the nuclear localization of desmin in early postnatal heart cells (Fig. 6D). The significant larger amounts of desmin in the cytoplasmic fraction of CMCs, as compared to stem cells, originates from a substantial increase in desmin expression in maturing CMCs and from the concomitantly occurring enormous increase in the cytoplasmic-to-nuclear volume ratio.

### mCherry-tagged desmin proteins are components of transcription factor complexes interacting with the *nkx2.5* gene in cardiac progenitor cells

To validate the interaction of desmin with the regulatory elements of the *nkx2.5* gene without using desmin-specific antibodies, ChIP analysis was performed in differentiating desmin-mCherry and mCherry-desmin-expressing ESC- and CVPCs-derived CPCs with a monoclonal mCherry specific antibody (Fig. 6E; Fig. S4E). Both mCherry-tagged desmin proteins bound to the MCE, the PE and to the promoter of the *nkx2.5* gene in a manner comparable to that of endogenous desmin. ChIP analysis in cell lines expressing only mCherry did not give any positive signal.

## DISCUSSION

In order to better understand the transcriptional regulation of cardiomyogenesis in cardiac progenitor cells (CPCs), we investigated the influence of the very early expressed muscle cell specific IF protein desmin on the transcription of the *nkx2.5* gene, one of the key regulators of cardiomyogenesis. This endeavor was based on the observation that desmin expression significantly enhanced Nkx2.5 expression and the development of beating cardiomyocytes (CMCs) in differentiating embryonic stem cells (ESCs). Using reporter-gene assays in different cell types and Nkx2.5 expression analysis in cardiac side population stem cells (CSPCs), combined with nuclear localization and ChIP assays, we demonstrate herein that desmin contributes to the transcriptional regulation of the *nkx2.5* gene in CPCs during a short period of cardiomyogenesis and in CSPCs of the adult mouse.

The present study strengthens the evidence of a functional interaction between desmin and the *nkx2.5* gene in CPCs, suggested by previous investigations (Hofner et al., 2007; Höllrigl et al., 2007) and provides evidence for a physical and functional interaction of desmin with the *nkx2.5* gene in CPCs for the first time. Furthermore, it suggests a physiologically relevant role of desmin for the regenerative capacity of the adult heart. Finally, it introduces Nkx2.5 as an additional factor potentially contributing to the worsening of the desmin knockout heart defects with increasing age and of human desmin related cardiomyopathies (Capetanaki et al., 2015; McLendon and Robbins, 2011; Milner et al., 1999).

Activation of nkx2.5-reporter transgenes by desmin in fibroblasts, myoblasts, and ESC-derived CPCs, in combination with its inhibition, mediated by desmin-siRNA in primary heart cells from newborn mice, as well as the reduced expression of Nkx2.5 in adult desmin null CSPCs (Figs 1–3), provide solid evidence for the involvement of desmin in the transcriptional regulation of the *nkx2.5* gene. Rescue of cardiomyogenesis in *nkx2.5^+/−^* haploinsufficient CPCs by desmin expression substantiates *in vivo* the direct positive influence of desmin on Nkx2.5 expression and on cardiomyogenesis in general. This subtle contribution of desmin to the transcriptional regulation in CPCs was not directly evident *in vivo* because desmin knockout mice did not show an obvious embryonic phenotype, most likely due to unknown redundant mechanisms safe-guarding cardiomyogenesis. However, reduced expression of Nkx2.5 in adult CSPCs lacking desmin might contribute to the severe defects observed in aging human and mouse hearts with mutant *desmin* alleles (Capetanaki et al., 2015). These very diverse defects might be co-founded by the inability of adult CSPCs lacking desmin or expressing dominant negative mutant desmin proteins to properly reactivate or up-regulate Nkx2.5 expression. The inadequate upregulation of Nkx2.5 expression in desmin null stem cells after injury or stress might contribute to a compromised regenerative potential in desmin-related heart diseases. The role of desmin as a co-factor acting in a dose-dependent manner upstream of *nkx2.5* is also supported by the previous finding that knock-down of desmin in C2C12 myoblasts and knockout in ESCs resulted in blocking myotube formation and the partial down-regulation of the *myoD* and *myogenin* genes (Li et al., 1994; Weitzer et al., 1995), which are key players of myogenic differentiation.

Activation of gene expression by desmin in a cell-autonomous manner requires either autocrine or mechanochemical activation of signal-transduction, sequestration of transcriptional inhibitors, or direct interaction with euchromatin in the nuclei of certain types of CPCs. Focusing on the latter, we were able to demonstrate the physical interaction of desmin with the minimal cardiac specific enhancer (MCE), the proximal enhancer (PE) and promoter region of the *nkx2.5* gene in CPCs by ChIP analysis (Figs 4 and 6; Table S2). Simultaneous binding of desmin to the distant MCE, the PE, and the promoter region of the *nkx2.5* gene with different affinity, and its ability to form dimers and tetramers (Quinlan et al., 1986) suggest a model wherein desmin might be part of different TF complexes bringing the distal MCE into proximity to the PE and the promoter region. By binding to three regulatory elements of the *nkx2.5* gene with different affinity, desmin may variably contribute to DNA bending and scaffolding TF complexes in front of the DNA-RNA polymerase II complex, and may by this way influence transcription. The significance of the desmin-*nkx2.5* gene interactions is also supported by the fact that it is only prominent at the beginning of cardiomyogenesis and is at/or below the detection limit in undifferentiated CVPCs, ESCs and fully developed CMCs. Furthermore, desmin was also present in TF complexes composed of nanog, brachyury, Mesp1, and Nkx2.5 in variable configuration. These TF complexes interact with the *nanog*, *brachyury*, *mesp1*, *nkx2.5*, and *desmin* genes in differentiating CPCs and thus allow the speculation on a potential broader modulating role of desmin in cardiomyogenesis.

The present data, however, do not discriminate between indirect and direct binding of desmin to the MCE and PEPR DNA. The former possibility is supported by the fact that the enhancer and promoter elements of the *nanog*, *brachyury*, *mesp1*, *nkx2.5*, and *desmin* genes, where desmin could be localized by ChIP, are also occupied by TFs nanog, brachyury, Mesp1, and Nkx2.5 (Table S2) (Bondue et al., 2008; David et al., 2011; Doppler et al., 2014). The latter possibility is supported by the fact that desmin also binds to a few DNA regions where none of the studied TFs were present, and by previous work demonstrating that desmin and other type III IFs directly interact with purified and synthetic DNA (Li et al., 2002, 2003; Tolstonog et al., 2005; Traub and Shoeman, 1994a,b). It will be a challenging task for future research to investigate the interaction of desmin with DNA at the molecular level in more detail.

Independently of the mode of interaction between desmin and DNA, ChIP data strongly suggest that low amounts of desmin must be present at least in some of the CPC nuclei at the beginning of cardiomyogenesis. Indeed, in differentiating ESCs and CVPC lines desmin was detected by confocal immunofluorescence microscopy in 7% and 10% of the cells, respectively. Notably, desmin was never detected in nuclei of undifferentiated ESC lines or in histological specimen from adult hearts. Absence of desmin in nuclei of mature CMCs is most likely due to the fact that desmin subunits are simply sequestered by abundantly emerging desmin-rich sub-cellular structures like Z-disks, costameres, and intercalated discs in addition to the IFs closely associated with mitochondria (Capetanaki et al., 2015). Preparation of nuclei from CPCs expressing mCherry-desmin fusion proteins allowed exclusion of the presence of any remnants of perinuclear localized desmin IFs by fluorescence microscopy and further demonstrated the nuclear localization of mCherry-tagged desmin proteins and desmin by western blot analysis with mCherry- and desmin-specific antibodies (Figs 5 and 6; Table 1).

Nuclear transport and presence of IF proteins has been demonstrated for tail-less cytokeratin 8, 18, and 19 in 3T3 fibroblasts (Bader et al., 1991), for vimentin in nasopharyngeal carcinoma lymph node metastasis (Luo et al., 2012), for nestin in various brain tumors (Krupkova et al., 2011), and for desmin in BHK21 cells (Kamei, 1986).

Desmin has no perfect nuclear localization signal in its carboxy-terminal domain like the lamins. However, it has a putative and suboptimal nuclear localization signal between amino acids R141 and R163 and a putative weak nuclear export signal located between amino acids L182 and L199 (NetNES 1.1 prediciton server, CBS) in its linker L1 and helix 1B domains. Alternatively, desmin may be transported to the nucleus by an unknown shuttle mechanism. A single study has suggested that vimentin may be transported to the nucleus by a cytoplasmic DNA-mediated transport mechanism (Hartig et al., 1998) and another, that desmin might be transported piggyback by filaggrin (Mack et al., 1993; Pearton et al., 2002). Finally, desmin may serve as a cytoplasmic docking site for nuclear proteins during the M-phase and in turn some of these proteins might facilitate the nuclear transport of desmin in a piggyback mechanism under certain circumstances in a small set of CPCs. Limitation of the nuclear presence of desmin and the desmin-chromatin interactions to a small subpopulation of CPCs during a short period of time at the beginning of cardiomyogenesis supports its specificity. Furthermore, these findings, together with the observation that even ubiquitous CMV-driven expression of mCherry-tagged desmin resulted in a nuclear localization in only 10% of nuclei of differentiating CVPCs suggest the existence of obligatory co-factors facilitating the nuclear transport of desmin and perhaps also its recruitment to the enhancer and promoter regions of the *nkx2.5* gene. Hence the major challenge for future investigations will be to unravel the mechanism of the nuclear transport of desmin in differentiating CPCs and to identify either the consensus DNA sequence or the chromatin components to which desmin binds in the nucleus of CPCs.

In summary, desmin, though not a typical TF may function as an auxiliary factor or scaffold with or without lamins in the nucleus, selectively modulating euchromatin, and thus permitting transcription of cardiac specific genes to proceed expeditiously and more efficiently.

## MATERIALS AND METHODS

### Cell culture

10T1/2 fibroblasts and C2C12 myoblasts were maintained in DMEM supplemented with 2 mM L-glutamine, 0.05 mg/ml streptomycin, 0.03 mg/ml penicillin, and 10% (v/v) fetal bovine serum (FBS) from Gibco (M10). Murine *des*^+/+^ (AB2.2 and W4) (Lauss et al., 2005), *des*^+/+^*des*^ect^ (DC6) (Hofner et al., 2007), *des*^−/−^ (Weitzer et al., 1995)*, des*^Δ1-48/Δ1-48^ (Höllrigl et al., 2002) ESCs and A5 cardiovascular progenitor cells (CVPCs) (Hoebaus et al., 2013) were maintained on SNL76/7 feeder cells in the same medium but supplemented with 15% (v/v) FBS (Hyclone, SH3007001) (M15) instead. Differentiation of ESCs and CVPCs was achieved by aggregation to embryoid bodies (EBs) and cardiac bodies as previously described (Hoebaus et al., 2013; Hofner et al., 2007).

### Isolation of cardiomyocytes

Primary cardiomyocytes (CMCs) were prepared from newborn Balb/C and C57BL/J6 mice (*Mus musculus* Linnaeus, 1758), respectively as previously described (Stary et al., 2005). Animal studies were conducted according to approved European, Austrian, and Greek Institutional animal guidelines (Directive 2010/63/EU). Heart tissue fragments were triturated three times in PBS containing 4% (v/v) pancreatin (Sigma, P3292) and 0.5 mg/ml collagenase II (Worthington, CLS-2) for 5 min, debris was removed by centrifugation, and lastly cardiac fibroblasts were removed by adsorption to a gelatin coated tissue culture plate for 45 min at 37°C. CMCs were maintained in DMEM supplemented with 2 mM L-glutamine, 0.05 mg/ml streptomycin, 0.03 mg/ml penicillin, and 4% (v/v) FBS (Sigma, F2442).

### Isolation, culture, FACS analysis, and quantitative RT-PCR of cardiac side population stem cells

Adult cardiac side population stem cells (CSPCs) were isolated from 8- to 12-weeks old 129SV wild type and *des^−/−^* mice (*Mus musculus* Linnaeus, 1758) as previously described (Pfister et al., 2010). Briefly, minced cardiac tissue (pool of six mouse hearts per genotype) was digested with 0.1% collagenase B (Roche, 11088815001), 2.4 U/ml Dispase II (Roche, 04942078001) and 2.5 mM CaCl_2_ in Hanks' balanced salt solution (HBSS) buffer supplemented with 2% FBS and 10 mM HEPES at 37°C for 30 min. Digestion was terminated by addition of HBSS buffer, digested tissue was filtered through a 70 μm filter, washed with HBSS buffer supplemented with 2% FBS and 10 mM HEPES and passed once more through a 40 μm filter. Total mono-nucleated cell number was determined and cells were re-suspended in high glucose DMEM (GIBCO, 41966-029), supplemented with 2% FBS and 10 mM HEPES at a concentration of 10^6^ cells/ml. Cells were stained with 2.5 μg/ml Hoechst 33342 (Sigma, B2261) at 37°C for 90 min with occasional shaking during incubation to ensure even staining. A negative control sample was also prepared by including 50 μM verapamil during the entire Hoechst staining procedure, in order to distinguish CSPCs. After staining, cells were washed twice with HBSS buffer supplemented with 2% FBS and 10 mM HEPES, resuspended in PBS w/o Mg^+2^/Ca^+2^, pH 7.4 (GIBCO, 70011-036) supplemented with 2% FBS and 2 mM EDTA and kept on ice until FACS analysis.

Freshly isolated CSPCs were cultured at a density of 2×10^5^ cells on 0.1% gelatine-coated 35×10 mm culture dishes in DMEM-F12 (GIBCO, 31330-038) supplemented with 10% FBS (GIBCO, 12484-028), 1% Penicillin/Streptomycin (GIBCO, 15070-063), 10 ng/ml basic fibroblast growth factor (Peprotech, 450-33), 20 ng/ml epidermal growth factor (Peprotech, 315-09), 1000 U/ml leukemia inhibitory factor (Sigma, L5158) and 1× insulin-transferrin-selenium stock (Invitrogen, 41400045), at 37°C, 5% CO_2_. Medium was renewed every three days and the resulting adherent cultures were passaged at 80% confluency using standard trypsinization techniques.

Fluorescence activated cell sorting of CSPCs was performed using a FACS AriaII-Upgraded (BD Biosciences) instrument equipped with triple lasers. The Hoechst dye was excited using a near UV laser (375 nm) and fluorescence emission was collected with a 450/20 nm filter (Hoechst Blue) and a 575/26 nm filter (Hoechst Red). A 502 nm long pass dichroic filter was used to separate the emission wavelengths. 7-amino-actinomycin D (7-AAD) was added to cells at a concentration of 0.25 μg/10^6^ cells immediately before sorting to exclude dead cells. Acquired data were analyzed using FlowJo software (FlowJo, LLC).

Total RNA was prepared from wild type and *des^−/−^* CSPCs, either freshly isolated or cultured for 10 days, with Tri Reagent (Sigma-Aldrich, T9424) according to manufacturer's instructions, with an additional DNase I treatment (Roche, 04716728001). cDNA was synthesized from 2 μg RNA using M-MLV reverse transcriptase (Invitrogen, 28025-013) and random primers (Invitrogen, 48190-011), according to manufacturers' instructions. The quantitative RT-PCR analysis (qRT-PCR) was performed from 5 ng cDNA with the LightCycler^®^ 480 Instrument (Roche) using the 480 SYBR Green I Master kit (Roche, 04707516001). The expression of *nkx2.5* RNA in wild type and *des^−/−^* CSPC was assayed in duplicate in five independent qRT-PCR experiments and was normalized to the expression of ribosomal S26 RNA (RPS26). The primers used for qRT-PCR amplification were as follows: *nkx2.5*: fwd: 5′-ACATTTTACCCGGGAGCCTA-3′, rev: 5′-GGCTTTGTCCAGCTCCACT-3′; RPS26: fwd: 5′-GCCATCCATAGCAAGGTTGT-3′, rev: 5′-GCCTCTTTACATGGGCTTTG-3′. The obtained data were expressed as mean±standard deviation (s.d.) of the fold expression of *nkx2.5* in *des^−/−^* versus wild type CSPCs.

### Generation and flow cytometry of *nkx2.5^+/EGFP^ des^+/+^* and *nkx2.5^+/EGFP^ des^+/+^ des^ect^* reporter ESCs

The plasmid pCSX-EGFP-PPDT (Hidaka et al., 2003) containing a *nkx2.5* transgene in which the exons have been replaced by the EGFP cDNA and a puromycin selection cassette, was introduced by homologous recombination into AB2.2 ESCs exactly as previously described (Höllrigl et al., 2001). The cells were selected with 1 µg/ml puromycin (Life Technologies) for four days, subcloned, tested for correct knock-in by PCR as described (Hidaka et al., 2003), and *nkx2.5^+/EGFP^ des^+/+^* ESC clones were tested for EGFP expression during differentiation of EBs by fluorescence microscopy.

To introduce an ectopic constitutive *desmin* allele under the control of the RSV promoter into *nkx2.5^+/EGFP^ des^+/+^puro^R^* ESCs, these cells were fused with *nkx2.5^+/+^des^+/+^des^ect^ neo^R^* DC6 ESCs (Hofner et al., 2007) using the polyethylene method. Cells selected with 1 µg/ml puromycin and 180 µg/ml active G418 (Gibco, 11811031) were sub-cloned and tested for EGFP and Desmin expression. Isolated clones not expressing desmin were used as controls for *in vitro* differentiation experiments.

Cells from EBs were separated by incubation with a mixture of trypsin solution (Invitrogen, 27250018), with 4% (v/v) pancreatin (Sigma, P3292) and 0.5 mg/ml collagenase (Whortington, CLS-2) for 20 min at 37°C two times and re-suspended in serum containing medium. Cells were harvested, resuspended in PBS, and EGFP fluorescence intensity was analyzed with a Becton & Dickinson LSR1 flow cytometer after elimination of dead cells positively stained for propidium iodide.

### Generation and analysis of ESC and CVPC lines expressing desmin-mCherry and mCherry-desmin fusion proteins

To generate red fluorescent mCherry-tagged desmin fusion proteins the murine desmin cDNA (NCBI Ref. Se.NP_034173.1) was amplified by PCR with primer pairs facilitating the in-frame insertion of the desmin cDNA either in front of the mCherry protein coding sequence into pmCherry-N1 or right after the mCherry coding region into pmCherry-C1 vectors (Clontech, 632523 and 632524). Synthesis and correct ligation of the CMV-promoter-driven mCherry-desmin and desmin-mCherry transgenes was verified by sequencing of both DNA strands. DraIII-linearized vector DNA was transfected into W4 ESCs and A5 CVPCs by electroporation as previously described (Höllrigl et al., 2001). After selection with 180 µg/ml G418 (GIBCO, 11811031) monoclonal cell lines were tested for mCherry-desmin and desmin-mCherry expression by fluorescence microscopy and western blotting.

### Confocal laser scanning fluorescence microscopy and image analysis

ESCs, CVPCs, and primary CMCs were processed and stained as previously described (Hofner et al., 2007). Briefly, cells were fixed in 4% paraformaldehyde in PBS for 20 min at room temperature, permeabilized with 0.15% saponin in PBS, and stained with antibodies against desmin (Santa Cruz, sc7559, 1:500, Sigma Aldrich, D1033, 1:50, and Abcam ab8592, 1:80), vimentin (Sigma, V6630, 1:200), cardiac troponin T (Thermo Scientific, MS-295, 1:200, and Santa Cruz, 20025, 1:200), emerin (Novocastra, NCL-Emerin; 1:50), and lamin A/C (Roblek et al., 2010; 1:500) for 60 min, washed three times with PBS, and stained with FITC- and TR-conjugated secondary antibodies (Dianova, 711-095-152, 1:200; 711-095-151, 1:200; 711-075-152, 1:200), and Alexa Fluor 488-conjugated secondary antibodies (Molecular Probes, A-11001, 1:1000) for 60 min. Alternatively, cells on cover slips were fixed in 96% ethanol at −20°C for 20 min, dried and blocked with 1% BSA in PBS for 10 min. Nuclei were counterstained with DAPI (Sigma, D9542). Desmin was scored to be intra-nuclear if the signal appeared and disappeared within the emerin-, lamin A/C-, or DAPI stained volume while scanning vertically through the nucleus. Spots at the periphery of nuclei were always excluded from the analysis (see Fig. 5H). For visualization of the mCherry-desmin, desmin-mCherry, and mCherry proteins, cells and nuclei, respectively, were fixed with 100% ethanol at −20°C for 5 min. Photomicrographs were taken on a Zeiss LSM 510 confocal microscope. 12, 16 or 25 *Z*-stacks of optically sectioned cells were obtained with a step size of 0.5 and 0.37 µm, respectively. Routine analysis was performed with an Axiovert 135TV microscope.

Nkx2.5-driven EGFP fluorescence in single living *nkx2.5^+/EGFP^ des^+/+^puro^R^* and *nkx2.5^+/EGFP^ des^+/+^des^ect^ neo^R^* cardiomyocytes were quantified by image analysis. Photomicrographs were taken on a Zeiss LSM 510 confocal microscope. Fluorescence images of single contracting cardiomyocytes were quantified with Adobe Photoshop software. To quantify EGFP fluorescence, images obtained with a 530 nm filter were imported into Photoshop CS2™ as tiff files and pixel intensity values from several areas of a cell were extracted by the built-in histogram tool. Pixel intensities were subtracted by the mean background pixel intensity obtained from several cytoplasmic areas of non-cardiomyocytes of the same image.

mCherry fluorescence images of nuclei were obtained from isolated nuclei taken from the fraction prepared for western blot experiments and nuclear fluorescence was quantified with Adobe Photoshop software. To quantify mCherry fluorescence, images obtained with a 615 nm filter were imported into Photoshop CS2™ as tiff files and pixel intensity values within several areas of cells and nuclei, respectively, were extracted by the built-in histogram tool. Pixel intensities were subtracted by background pixel intensity obtained from several cell- or nucleus-free areas of the images.

### Chromatin immunoprecipitation and PCR analysis

Preparation of soluble chromatin and ChIP assays were carried out as previously described (Hauser et al., 2002). Shortly, ESCs, CVPCs and primary CMCs were fixed using formaldehyde and lysed with a buffer containing 10% SDS, Roche complete protease inhibitor cocktail, and PMSF. Equal amounts of chromatin were sonicated, and DNA-protein complexes were immuno-precipitated over night with antibodies against desmin (Abcam, ab8592, Sigma, D8281 and D1030), brachyury (Santa Cruz, sc17743 and sc17745), and mCherry (St. John's Laboratory, STJ34373), respectively. As positive and negative controls, antibodies against the C-terminus of histone H3 (Abcam, ab7228), c-Myc (AbCam, 1791), vimentin (Sigma, V4630), and IgG (Santa Cruz, sc2027) were used. Using pre-immune IgG as a negative control did not result in any signals above the limit of detection. ChIP DNA and control input DNA were analyzed by PCR, using 35 to 40 cycles and primer pairs (for sequences see Table S1) situated in the 5′-region of the murine *nkx2.5* locus (NCBI: Gene ID: 18091, updated on 14 July 2015) as indicated in Fig. 4A and Fig. 6E. For location of primer pairs used to analyze the interaction of nanog (antibodies: Santa Cruz, sc33760; Cell Signaling, 8822), brachyury (antibody: Santa Cruz, sc17745), mesp1 (antibody: Santa Cruz, sc1763078), nkx2.5 (antibodies: Santa Cruz, sc8697 and 14033), and desmin (antibodies: Abcam, ab8592; Sigma, D8281 and D1030), with the *nanog*, *brachyury*, *mesp1*, *nkx2.5*, and *desmin* gene, see Fig. S5. Quantification was performed by measuring the luminosity of ethidium bromide stained PCR products from at least two independent experiments by Adobe Photoshop CS2™ tools.

### Reporter gene assays

10T1/2 and C2C12 cells were transiently transfected by the classical calcium-phosphate method with a DMSO shock, and primary CMCs with the Lipofectamine 2000 (Invitrogen, 11668-019) method. DNA for transfection was isolated with the Endo Free Plasmid Maxi Kit (Qiagen, 12263) from the following plasmids: pGL3b, containing the *Photinus pyralis* luciferase (LUC) cDNA (Promega, E1751) and phRL-TK (Promega, E2241), containing the *Renilla reniformis* luciferase cDNA as reporter and transfection efficiency control; pNKE24 (Searcy et al., 1998), a pGL3b backbone, containing the *nkx2.5* proximal enhancer and promoter region (PEPR), located between base pairs −3059 to −2554 of the *nkx2.5* 5′-region; pMCE, containing the minimal cardiac specific enhancer (MCE) located between base pairs −9432 to −8922 of the *nkx2.5* 5′-region (Lien et al., 1999), in addition to the PEPR in the LUC reporter plasmid; pRSV-desmin (‘desmin-ect.’), containing the desmin cDNA under the control of the RSV promoter (Hofner et al., 2007); pshRNA desmin knock-down plasmids (‘des-shRNA’), which performed equally well in reducing *desmin* mRNA levels; clone 1: 5′-TCCTACACCTGCGAGATTGAT-3′; clone 2: 5′-GATCGCGTTCCTTAAGAAAGT-3′; clone 3: 5′-CAAGGGCTCCTCGAGTTCAAT-3′; clone 4: 5′-AGACCATCGCGGCTAAGAACAT-3′; pshRNA-neg (‘nc-shRNA’): 5′-GGAATCTCATTCGATGCATAC-3′, as negative control (SA Biosciences); and pUC19, for leveling the concentration of transfected DNA.

The pMCE LUC reporter plasmid was generated by cloning the MCE into the SalI restriction site of pNKE24. This resulted in the location of the MCE upstream of base pair −2641, 5′ to the PEPR in the pNKE24 plasmid. PCR primers were designed for the production of the MCE insert from C3H mouse genomic DNA, with flanking SalI restriction sites: SalI fwd: 5′-TAATGTCGACTCTGGGTCCTAATGC-3′and SalI rev: 5′-TATTGTCGACTCAAGGTGCACATGA-3′. The PCR product was generated using *Pyrococcus furiosus* polymerase (Thermo Scientific, EP0501) and both strands were sequenced to verify accurate synthesis.

Transfected cells were incubated in M10 medium for 48 h and then lysed and firefly and renilla LUC activity was measured with the Dual Luciferase^®^ Reporter System (Promega, E1910) using a Centro LB 960 luminometer (Berthold Technologies). *Renilla reniformis* LUC activity from co-transfected phRL-TK plasmids was used to correct firefly LUC activity for varying transfection efficiency. LUC activity was always normalized to the basic activity of the promoter-less pGL3b plasmid. Expression of *desmin* in cells transfected with pshRNA-anti-Desmin clones 1-4 and pshRNA-neg was quantified by RT-PCR.

mRNA was isolated from transfected 10T1/2 and C2C12 cells with the Qiagen RNeasy kit. cDNA was synthesized with RevertAid M-MuLV reverse transcriptase (Thermo Scientific, EP0441). RT-PCR was performed with Taq polymerase (Thermo Scientific, EP0402) using primers for desmin as follows: fwd: 5′-TGATGAGGCAGATGAGGGAG-3′, rev: 5′-TGAGAGCAGAGAAGGTCTGG-3′. Amplification conditions: Tm: 52°C, 35 cycles. Quantification was performed by measuring the luminosity of ethidium bromide stained RT-PCR products by Adobe Photoshop CS2™ tools of two independent experiments and four RT-PCR analyses.

### Isolation of nuclei and chromatin associated proteins and western blot analysis

To obtain cytoplasm-free nuclei from cells undergoing cardiomyogenesis a high-quality biochemical fractionation protocol (Rosner et al., 2013) was employed with some modifications allowing the separation of the nuclear membranes from the chromatin (Aaronson and Blobel, 1975). Cells from 6.7-day old EBs and 9.3-day old cardiac bodies, respectively, expressing either mCherry, the desmin-mCherry, or the mCherry-desmin fusion protein, were dissociated by incubation with a mixture of trypsin solution (Invitrogen, 27250018), with 0.1% (m/v) pancreatin (Sigma, P3292) and 0.05% (m/v) collagenase type 2 (Whortington, CLS-2) at 37°C for 25 min. Cells were suspended in M15, cell aggregates removed by low-speed centrifugation, and single cells were recovered by centrifugation. Cells were washed once with ice cold PBS and then carefully lysed in 5× the pellet volume of 20 mM Tris-HCl pH 7.6, 50 mM β-mercaptoethanol, 0.1 mM EDTA, 2 mM MgCl_2_, 174 µg/ml PMSF (Sigma, P7626), 2 µg/ml leupetin (Sigma, L2884), 2 µg/ml aprotinin (Sigma, A6279), and 0.3 µg/ml benzamidine chloride (Sigma, B6506) (solution 1) at room temperature for 2 min and then at 4°C for 10 min. After adding 10% (v/v) of a 10% Nonidet-P40 (Sigma 74385) solution to the lysates, cell fragments were carefully suspended 3× with a 200 µl Gilson tip and nuclei were pelleted at 600×***g*** and 4°C for 5 min. The supernatant containing the cytoplasmic fraction of the cells was removed and nuclei were washed to remove all cytoplasmic components from their surface. To maintain the integrity of the nuclei, they were carefully suspended 3× with a 200 µl Gilson tip in 0.5 ml of solution 1 containing 1% Nonidet-P40 and then pelleted at 600×***g*** and 4°C for 5 min. This procedure was repeated twice and purity of nuclei was assessed by fluorescence microscopy. After removal of the last wash solution, nuclei were lysed in 15× the pellet volume of 6 mM NaH_2_PO_4_ pH 7.1, 603 mM KCl, 171 mM NaCl, 1% Triton X-100, 8 mM β-mercaptoethanol, and 348 µg/ml PMSF in a micro douncer at 4°C for 1 h. Insoluble chromatin was pelleted at 17,000×***g*** at 4°C for 25 min, the supernatant containing the karyoplasm was removed, and the chromatin solubilized in 1× SDS sample buffer (Laemmli, 1970) containing 1 mg/ml DNase I (Boehringer, 104159) by repeated pipetting at room temperature and final incubation at 96°C for 5 min. Samples of the cytoplasmic, karyoplasmic, and chromatin fraction were stored in 1× SDS sample buffer at −20°C.

Western blot analysis of the samples was performed by standard procedures with antibodies against mCherry (St. John's Laboratory, STJ34373), desmin (Abcam, ab8592 and Sigma, D8281), histone H3 (Abcam, ab7228), and gp130 (Santa Cruz, sc656), respectively, and secondary-alkaline phosphatase-conjugated antibodies (Promega S372B, S373B, and V1151).

### Statistical analysis

All data are presented as the arithmetic mean±s.d. σ_x(n−1)_. Statistical significance was evaluated using Student's *t*-test and values of *P*≤0.05 were considered to indicate statistical significance.
